# Anthrax Lethal Toxin Disrupts Intestinal Barrier Function and Causes Systemic Infections with Enteric Bacteria

**DOI:** 10.1371/journal.pone.0033583

**Published:** 2012-03-16

**Authors:** Chen Sun, Hui Fang, Tao Xie, Roger D. Auth, Nayana Patel, Patrick R. Murray, Philip J. Snoy, David M. Frucht

**Affiliations:** 1 Laboratory of Cell Biology, Division of Monoclonal Antibodies, Office of Biotechnology Products, Center for Drug Evaluation and Research, United States Food and Drug Administration, Bethesda, Maryland, United States of America; 2 Department of Laboratory Medicine, Warren Magnusen Clinical Center, National Institutes of Health, Bethesda, Maryland, United States of America; 3 Division of Veterinary Services, Center for Biologics Evaluation and Research, United States Food and Drug Administration, Bethesda, Maryland, United States of America; Charité-University Medicine Berlin, Germany

## Abstract

A variety of intestinal pathogens have virulence factors that target mitogen activated protein kinase (MAPK) signaling pathways, including *Bacillus anthracis*. Anthrax lethal toxin (LT) has specific proteolytic activity against the upstream regulators of MAPKs, the MAPK kinases (MKKs). Using a murine model of intoxication, we show that LT causes the dose-dependent disruption of intestinal epithelial integrity, characterized by mucosal erosion, ulceration, and bleeding. This pathology correlates with an LT-dependent blockade of intestinal crypt cell proliferation, accompanied by marked apoptosis in the villus tips. C57BL/6J mice treated with intravenous LT nearly uniformly develop systemic infections with commensal enteric organisms within 72 hours of administration. LT-dependent intestinal pathology depends upon its proteolytic activity and is partially attenuated by co-administration of broad spectrum antibiotics, indicating that it is both a cause and an effect of infection. These findings indicate that targeting of MAPK signaling pathways by anthrax LT compromises the structural integrity of the mucosal layer, serving to undermine the effectiveness of the intestinal barrier. Combined with the well-described immunosuppressive effects of LT, this disruption of the intestinal barrier provides a potential mechanism for host invasion via the enteric route, a common portal of entry during the natural infection cycle of *Bacillus anthracis*.

## Introduction

*Bacillus anthracis* (anthrax) infection most commonly occurs in herbivores, which in the natural infection cycle consume infectious spores that are present in contaminated soils [1]. *Bacillus anthracis* carries an essential virulence plasmid, pX01, that encodes the three components of anthrax toxin: edema factor (EF), lethal factor (LF), and the host receptor-binding protective antigen (PA) [2]. PA facilitates intracellular delivery of EF, an adenylate cyclase, and/or LF, a metalloprotease with specific activity against MAPK kinases (MKKs) [3]. The effects of the administration of the combination of PA and LF, termed lethal toxin (LT), have been the subject of intense research interest, because LT reproduces many of the clinical features of anthrax infection in animal models [4], [5].

As anthrax is generally a gastrointestinal infection in the natural setting [1], it would be predicted that this pathogen has evolved mechanisms to facilitate infection via this route. In common with the enteric pathogens, *Shigella flexneri*, *Yersinia enterocolitica* and *Salmonella enterica*, *Bacillus anthracis* has virulence factors that target components of the MAPK signaling pathways [6], [7], [8], [9]. This shared strategy of enteric pathogens would suggest that the targeting of MKKs by LT might represent a mechanism to promote infection via the gastrointestinal route of entry.

Previous studies addressing the effects of LT on the intestine generated conflicting results. Nearly a decade ago, fecal blood was reported in the intestines of occasional, LT-treated mice, however there was no microscopic evidence of intestinal pathology [10]. A recent study by the same group reported multifocal intestinal ulcerations in the setting of immunocompromised MyD88-deficient mice treated with LT, but not in heterozygous animals, which were reported to have minimal incidence of intestinal ulceration [11]. In contrast, our findings demonstrated marked ulceration and hemorrhage in wild-type C57BL/6J mice treated with LT [12]. This apparent contradiction presented a compelling basis for additional investigation to clarify whether LT mediates pathogenic effects in the intestines of immunocompetant hosts, a question potentially relevant to pathogenesis in the natural anthrax infection cycle.

Using a series of experiments involving histological and microbiological assessments, we have extensively characterized the effects of LT on intestinal tissues. At a high dose of intravenous LT, mice develop intestinal ulcerations and bleeding; these effects depend upon the proteolytic activity of its LF component. LT-induced intestinal pathology is distinguished by a blockade in epithelial progenitor cell proliferation, accompanied by the marked enhancement of apoptosis in the villus tips. We herein report that this intestinal pathology is associated with a breakdown in the host intestinal barrier, as nearly all wild-type C57BL/6J mice and a substantial fraction of BALB/c mice treated with high-dose LT develop systemic infections with enteric organisms within 72 h of exposure. This effect is at least as rapid as the development of infectious complications reported following radiation or chemotherapy [13], [14], [15], [16]. These findings indicate that targeting of MKKs by anthrax LT results in severe compromise of the intestinal barrier in immunonocompetant hosts, suggesting a potential mechanism for bacterial entry via the enteric route.

## Results

### Anthrax LT induced intestinal pathology is not route- or strain-dependent

**Figure 1:**
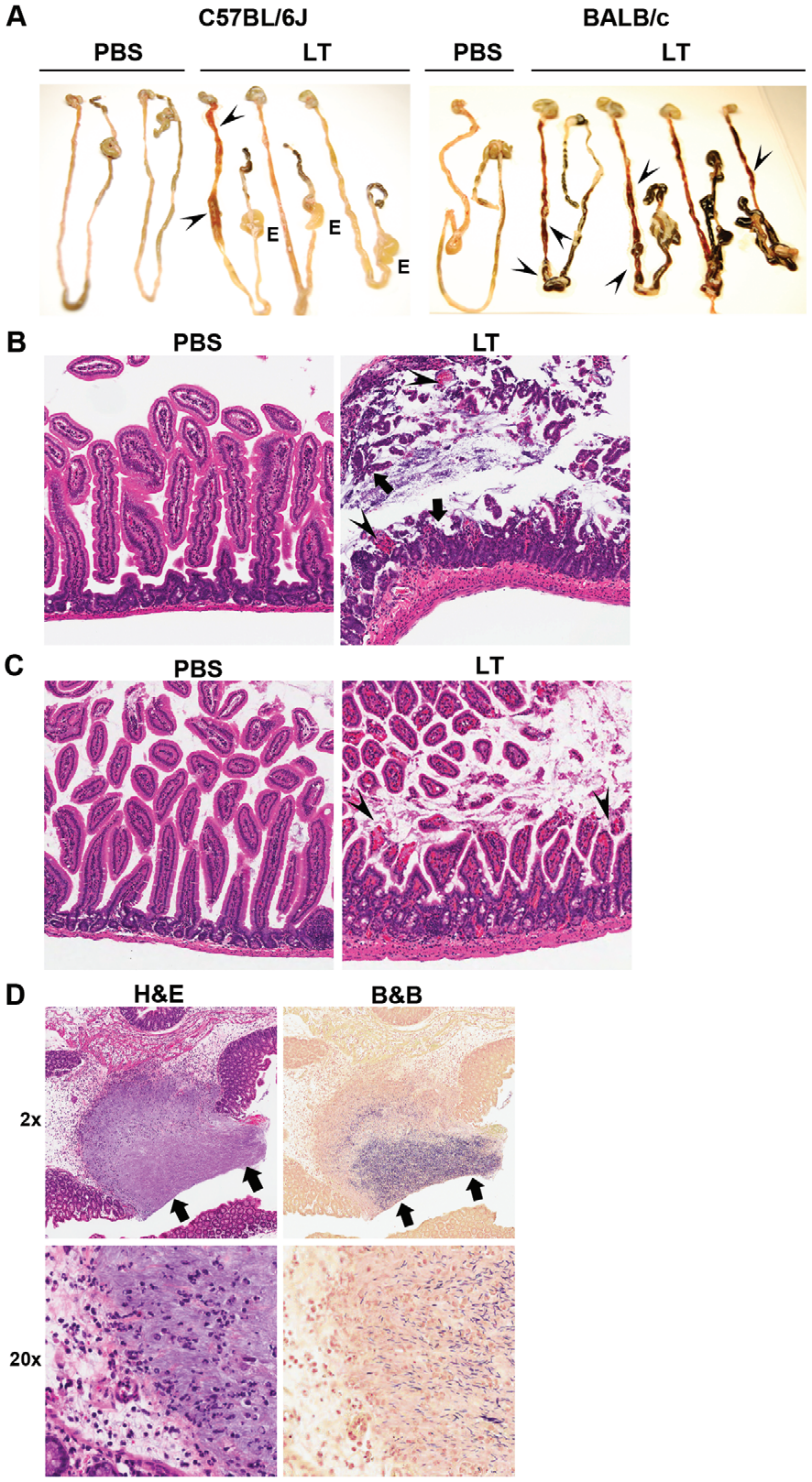
Anthrax LT causes intestinal damage in C57BL/6J and BALB/c mice. **(A) C57BL/6J and BALB/c mice were injected intravenously with anthrax LT or the PBS vehicle alone.** Intestines obtained from moribund (LT-treated) or control (PBS-treated) animals were photographed and assessed for gross pathological changes. Arrowheads indicate areas of hemorrhage. Areas of edema are marked by the letter “E”. (**B** and **C**) C57BL/6J and BALB/c mice were injected intravenously with anthrax LT (n = 34 and n = 14, respectively) or the PBS vehicle alone (n = 15 and n = 10, respectively). LT-treated mice were sacrificed when they became moribund; PBS-treated mice were sacrificed simultaneously as controls. Shown are representative H&E sections from the small intestines of LT- and PBS-treated C57BL/6J mice (**B**, n = 34), and BALB/c mice (**C**, n = 14). All mice within each strain displayed similar histological findings. Arrows indicate mucosal erosions/ulcerations, and arrowheads identify areas of hemorrhage. Aperio ScanScope-acquired images are shown at 5×. (**D**) Shown are adjacent sections from a typical ulcer in a moribund LT-treated C57BL/6J mouse stained with H&E (left panels) or Brown & Brenn (right panels). High magnification reveals the submucosal penetration of bacteria. Aperio ScanScope-acquired images are shown at 2× or 20×.

We recently reported that wild-type C57BL/6J mice administered intraperitoneal LT develop marked multi-focal ulcerations in the small intestine [12]. To confirm that our findings were not route- or strain-dependent, we administered intravenous LT to both C57BL/6J and BALB/c mice. Pathological samples obtained from moribund animals revealed evidence of gross intestinal bleeding in both strains of mice; however, C57BL/6J mice exhibited more intestinal edema following LT treatment (**Figure 1A**, left panel). In contrast, BALB/c mice showed greater amounts of gross bleeding than C57BL/6J mice (**Figure 1A**, right panel).

Despite some gross pathological differences, histological examination revealed similarities between the two strains. Mice that became moribund and/or succumbed to LT within 48 h post-administration showed little evidence of intestinal ulceration (not shown), whereas animals that survived beyond this point exhibited a distinct pattern of damage, characterized by multi-focal intestinal erosions and ulcerations, with associated bleeding and abscess formation (**Figures 1B–D**). Preparations of adjacent sections stained using Brown & Brenn revealed penetration of bacteria into the submucosa (**Figure 1D**). Although LT-treated C57BL/6J and BALB/c mice shared pathological features, sections from LT-treated BALB/c mice generally showed more evidence of hemorrhage (**Figure 1C**), corresponding with gross histological findings (**Figure 1A**). Sections from LT-treated C57BL/6J mice, in contrast, showed evidence of more severe and widespread ulcer formation (**Figure 1B**).

### Time-course progression of anthrax LT-induced intestinal pathology

**Figure 2:**
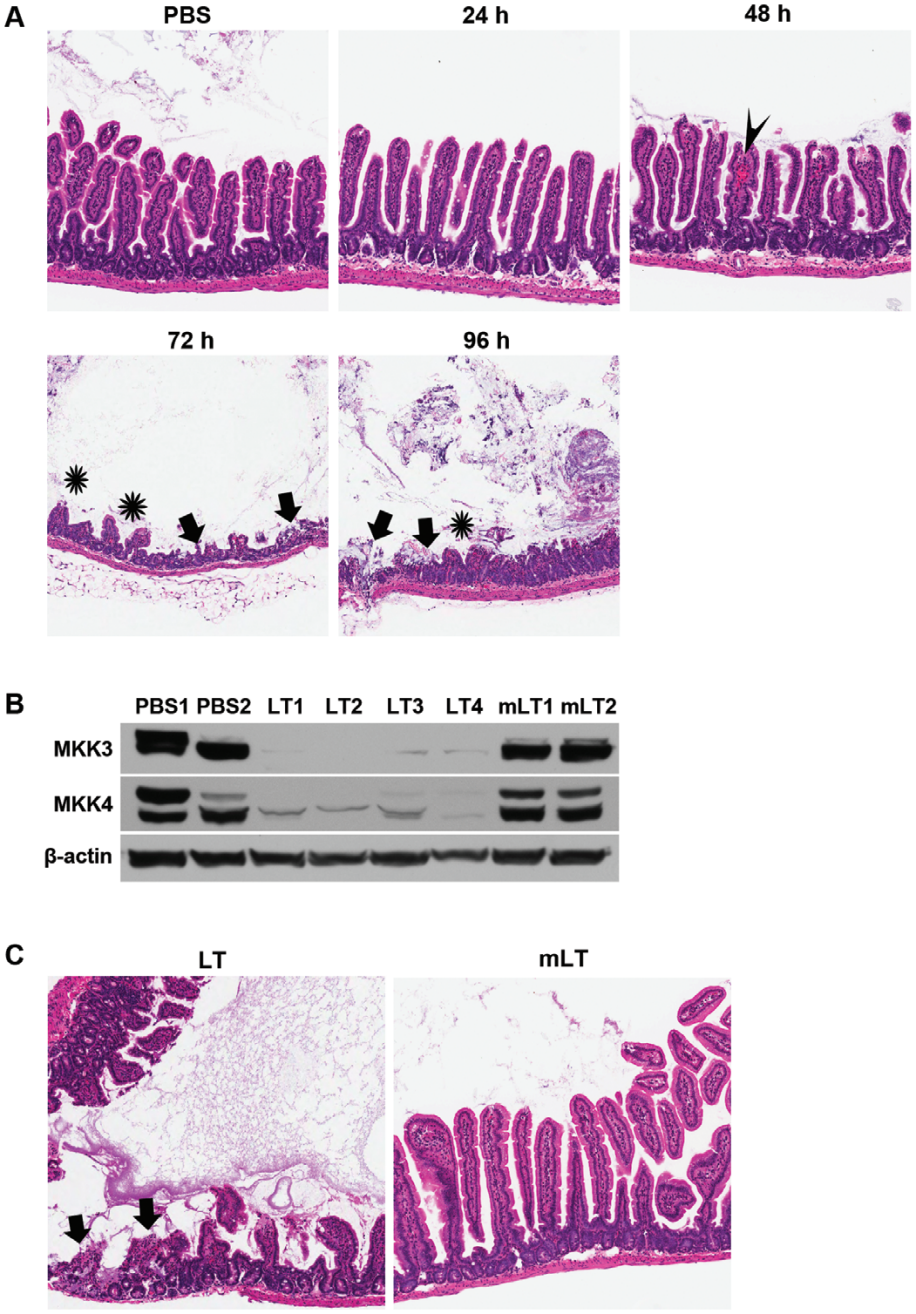
Pathological effects of LT progress in a time-dependent manner following exposure and require the enzymatic activity of LF. (**A**) C57BL/6J mice were euthanized at the indicated time points following intravenous LT treatment (24–96 h, 20 animals/cohort), along with control animals that received the PBS vehicle. Representative H&E-stained sections from the small intestines of these animals are shown; all mice in each cohort showed similar histological findings. Aperio ScanScope-acquired images are shown at 5×. Arrowheads indicate hemorrhage, asterisks indicate villous blunting, and arrows indicate mucosal erosion/ulceration. (**B**) C57BL/6J mice were injected intravenously with LT (LT 1–4), LT comprising wild-type PA and a protease-deficient LF mutant (mLT 1–2), or the PBS vehicle (PBS 1–2). Protein levels of MKK3 and MKK4 in intestinal tissue samples were analyzed by western blotting 24 h post administration is shown. β-actin protein levels were assessed to demonstrate loading. (**C**) C57BL/6J mice were injected intravenously with LT (n = 5) or mLT (n = 5). Mice that received LT alone showed signs of intoxication 48–96 hours post-treatment, whereas those that received mLT showed no signs of intoxication. When moribund, LT-treated mice were euthanized, simultaneously with mLT-treated control mice. Shown are representative H&E sections from LT-treated (left panel) and mLT-treated mice (right panel); all mice in each cohort showed similar histological findings. Arrows indicate areas of mucosal erosion/ulceration.

BALB/c mice succumb rapidly in response to LT, as they carry a sensitive allele of Nalp1b that promotes caspase-1 activation and inflammatory cytokine release in response to LT, whereas C57BL/6J mice carry a resistant Nalp1b allele [17]. For this reason, BALB/c mice are more likely than C57BL/6J mice to succumb to LT within the first 48 h following toxin administration, prior to developing intestinal pathology. To better understand how LT mediates pathological effects in the intestine, we used C57BL/6J mice in the subsequent experiments to avoid early animal deaths. In a time-course study (**Figure 2A**), LT-induced pathological changes became evident in the intestine 48 h post LT exposure. These changes included the onset of villous damage and minor hemorrhage. By 72 h post LT exposure, focal areas showed marked destruction of the normal villous structures, accompanied by areas of ulceration. This pathology persisted in the few animals that survived to 96 h post exposure. The pathological effects of LT on the intestinal barrier were mediated by the proteolytic activity of its LF component on MKKs, as demonstrated by mice administered LT comprising wild-type PA and protease-deficient LF (mLT). In contrast to mice receiving wild-type LT that showed disrupted MKK signaling and intestinal ulceration, mice receiving mLT showed no defect in MKK signaling pathways and no pathological or histological evidence of intestinal damage (**Figures 2B and 2C**).

### Anthrax LT induces systemic infections with enteric bacteria in wild-type mice

The first indication that LT-treated mice might be developing systemic bacterial infections was the observation that mice treated with LT developed severe hypoglycemia with serum glucose levels dropping precipitously prior to death (**Figure S1**). One potential explanation for the hypoglycemia in LT-treated animals was that it was secondary to the effects of systemic bacteremia [18], [19]. To investigate this possibility, we performed abdominal wash and blood cultures on samples obtained from moribund LT-treated C57LB/6J and BALB/c mice. Ninety-four percent of C57BL/6J animals (32/34) had developed systemic bacterial infections, with 29/34 animals becoming bacteremic (**Table 1**). Systemic bacterial infections were also observed in 5/14 LT-treated BALB/c mice (**Table S1**). BALB/c mice are very sensitive to LT [17], and half of the mice (7/14) in our experiment died within 48 h of LT exposure. None of these animals developed systemic bacterial infection. However, a majority of the mice (5/7) that survived longer than 48 h post LT exposure (52–66 hours) developed systemic bacterial infections. In addition, three moribund animals in each strain were culture positive for abdominal cavity infections in the absence of bacteremia (6 animals total), whereas only one C57BL/6J mouse was bacteremic in the absence of abdominal cavity infection (**Table 1** and **Table S1**), suggesting that the abdominal cavity was the primary site of infection prior to hematological dissemination. LT-induced infection required the proteolytic activity of its LF component; mice administered LT with a protease-deficient LF component neither showed intestinal MKK cleavage *in vivo* (**Figure 2B**) nor developed bacterial infections (**Table S2**).

**Table 1:** Bacterial Culture Results in LT-treated Mice at Autopsy (C57BL/6J).

	Bacterial Culture Results (n = 34)	
N	Abdominal cavity	Blood
2	**−**	**−**
3	**+**	**−**
1	**−**	**+**
28	**+**	**+**

Next, we used an automated bacterial identification system, supplemented with conventional biochemical tests, to identify the bacterial species causing the underlying infections. These studies revealed that the infections were caused by enteric flora, with *Enterococcus faecalis*, *Enterobacter cloacae*, and *Escherichia coli* accounting for over 70% of the isolates (**Table 2**). We then investigated the development of systemic infections in time-course experiments following exposure to LT (**Table 3**). Peritoneal and blood culture results were negative in PBS-treated controls and in mice treated with LT for 24 h prior to sampling. At 48 h post exposure, 4/15 animals exhibited bacterial growth in cultures of peritoneal washes, whereas only 1/15 animals had a positive blood culture. Animals euthanized 72 h and 96 h post exposure had nearly uniformly positive blood and peritoneal wash cultures. These findings closely correlated with the time-course of the intestinal pathology observed in LT-treated mice and were consistent with conclusion that peritoneal infection generally precedes hematological dissemination.

**Table 2:** Bacterial Blood Culture Results in LT-treated Mice*.

Species	N
Enterococcus faecalis	22
Enterobacter cloacae	15
Escherichia coli	12
Staphylococcus species	8
Klebsiella oxytoca	6
Acinetobacter	6

* Moribund, LT-treated C57BL/6J mice; n = 34 total; some mice were co-infected with 2–3 bacterial species.

**Table 3:** Bacterial Culture Results at Autopsy (C57BL/6J Mice).

	Time course of LT treatment				
	PBS (n = 15*)	24 h (n = 15)	48 h (n = 15)	72 h (n = 15)	96 h (n = 15)
Culture positive in abdominal cavity	0	0	4	10	14
Bacteremia	0	0	1	9	14

* PBS-treated control mice were sacrificed at varying time points following administration: 24 h (n = 3), 48 h (n = 3), 72 h (n = 3), 96 h (n = 6).

### The pathological effects of anthrax LT on the intestine are dose-dependent

**Figure 3:**
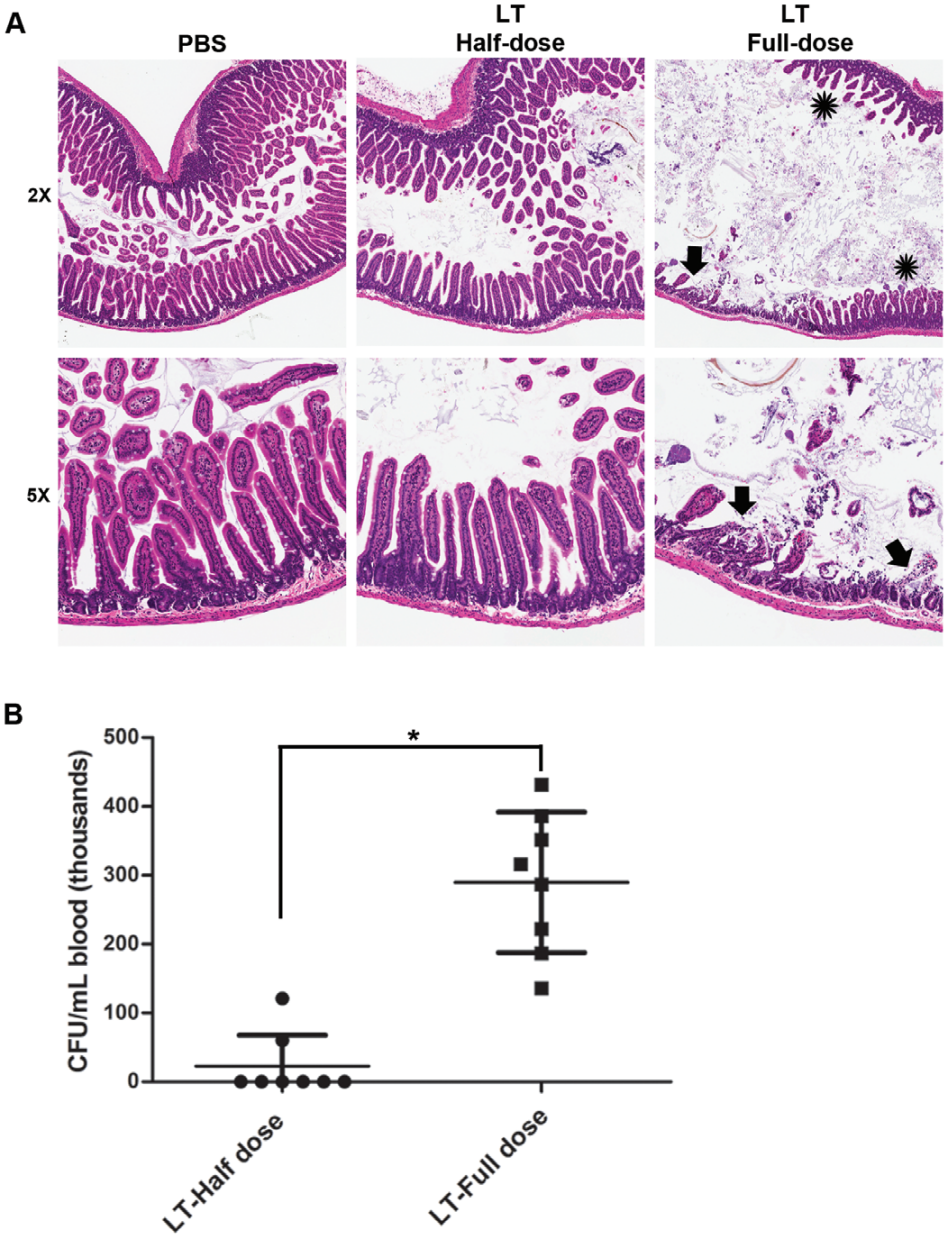
Dose-dependent effects of anthrax LT on the intestinal barrier. (**A**) C57BL/6J mice were injected intravenously with PBS (control, n = 5), 100 µg PA/40 µg LF (n = 10), or 200 µg PA/80 µg LF (n = 10). LT-treated animals were euthanized when they became moribund, each with a simultaneously euthanized PBS-treated control. Samples from the small intestines of these animals were analyzed by H&E staining. Representative sections from a control animal and animals from each of the two LT-dose cohorts are shown; all animals in each cohort showed similar histological findings. Aperio ScanScope-acquired images are shown at 5×. Arrows indicate mucosal ulcerations and asterisks identify the area of villous blunting. (**B**) C57BL/6J mice were injected intravenously with 200 µg PA/80 µg LF (n = 8) or 100 µg PA/40 µg LF (n = 8) as shown. Cardiac blood samples were collected when the mice became moribund. Bacterial count data are shown (mean ± SD; * p<0.001, Student's t-test).

To resolve potential inconsistencies in the literature, we explored the contribution of toxin dose to the development of intestinal pathology. We routinely used a dose of 200 µg PA and 80 µg LF for experiments with C57BL/6J mice, representing a 2.7 molar ratio of PA/LF that slightly exceeds the 7∶3 molar ratio of the fully occupied toxin complex. Our dosing regimen differed with that used by the other group that reported minimal LT-induced pathology in the intestines of wild-type or heterozygous MyD88 ^+/−^ mice (100 µg PA and 100 µg LF) [10], [11]. We hypothesized that our regimen could represent a higher effective dose than the 1∶1 weight-based ratio used by the other group. Therefore, we investigated a lower dose to determine whether we could explain the findings of these other reports. These experiments revealed that mice that received a reduced dose of LT (100 µg PA and 40 µg LF) still developed lethal intoxication, but deaths in these animals were delayed relative to mice receiving the higher dose (>1 wk vs. ≤4 d post-treatment, data not shown). In addition, there was little evidence of intestinal pathology in mice receiving the reduced LT dose (**Figure 3A**). Moreover, most mice in the lower dose group were blood culture negative, whereas all of the animals receiving the full dose developed systemic infections with relatively high levels of circulating bacteria (**Figure 3B** and **Table S3**). Thus, LT-induced pathology and the development of systemic bacterial infections is dose-dependent, an observation that could explain apparent discrepancies in the literature.

### Broad spectrum antibiotic therapy attenuates pathology in LT-treated mice

**Figure 4:**
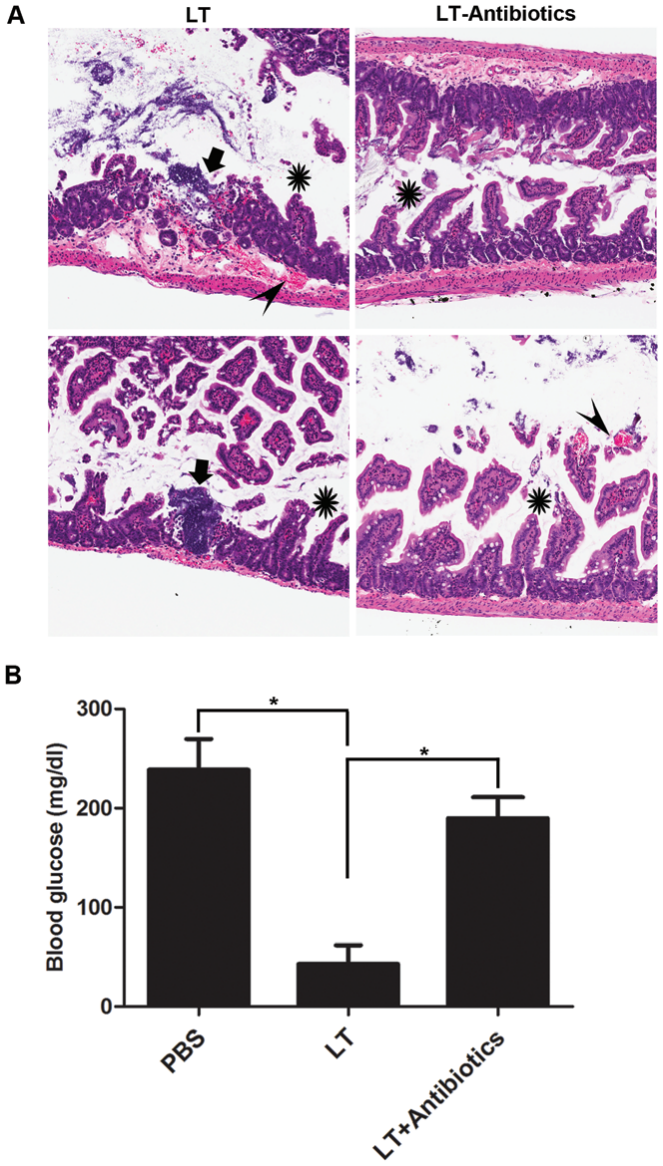
Administration of antibiotics partially attenuates intestinal pathology in LT-treated mice. C57BL/6J mice were administered either LT alone or LT with concomitant amoxicillin and gentamicin. Mice were euthanized when they became moribund, which occurred at the same time frame for both cohorts (data not shown). (**A**) Samples from the small intestine were obtained and analyzed by H&E staining, as shown in representative sections from 2/15 animals from each cohort. All mice in each cohort showed similar histological findings. Arrowheads indicate hemorrhage, asterisks indicate villous blunting, and arrows indicate mucosal erosions/ulcerations. Aperio ScanScope-acquired images are shown at 5×. (**B**) Blood samples obtained by cardiac puncture were assessed for glucose concentrations. (n = 15 for each cohort, * p<0.0001, Student's t test).

Therapy with gentamicin and amoxicillin was used to assess the role of bacterial infection in mediating the effects of LT, as this antibiotic combination provided broad coverage against the enteric organisms cultured from the abdominal cavities and blood of LT-treated mice. Whereas all the LT-treated control animals developed bacteremia, only 1/15 animals receiving antibiotics developed a positive abdominal wash culture, and none developed bacteremia (**Table S4**). The co-administration of broad spectrum antibiotics did not prevent LT-induced structural damage to the villi. Antibiotic treatment did, however, attenuate villous blunting and reduce the size and frequency of the associated intestinal erosions and ulcerations (**Figure 4A**). This suggested a role for invading bacteria in enhancing some, but not all, of the features of intestinal damage. Moreover, the administration of antibiotics reversed the hypoglycemia observed in moribund, LT-treated mice (**Figure 4B**). This finding was consistent with our hypothesis that LT-induced hypoglycemia was secondary to systemic bacterial infection.

### Anthrax LT has anti-proliferative and pro-apoptotic effects on the intestinal epithelium

**Figure 5:**
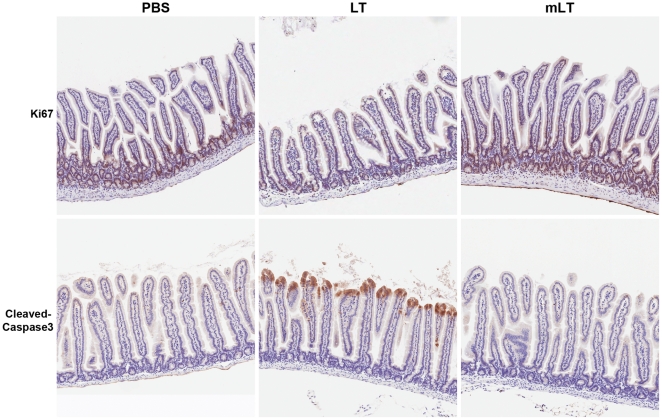
Anthrax LT blocks intestinal epithelial cell proliferation and promotes apoptosis. C57BL/6J mice were euthanized 48 h following the administration of LT (n = 5), protease-deficient mLT, or PBS (n = 5, controls). (**A**) Sections from the small intestines of these animals were evaluated by immunohistochemistry for the Ki67. Representative sections from damage-free areas of PBS-treated, LT-treated, and mLT-treated animals are shown in the upper panels for comparison. All animals in each cohort showed similar histological findings. Aperio ScanScope-acquired images are shown at 5×. (**B**) Sections from the small intestines of these animals were evaluated by immunohistochemistry for activated caspase-3. Representative sections from damage-free areas of PBS-treated, LT-treated, and mLT-treated animals are shown in the lower panels for comparison; all animals in each cohort showed similar histological findings. Aperio ScanScope-acquired images are shown at 5×.

As LT is known to have anti-proliferative effects on a broad spectrum of cell types [4], we hypothesized that it could have this effect on intestinal epithelial cells as well. Intestinal sections from mice treated with LT or PBS control for 48 h were stained with anti-Ki67, a marker of cellular proliferation. Ki67 staining was observed in the crypts of the villi in sections from PBS-treated control animals (**Figure 5**, upper left panel), the location of intestinal epithelial progenitor cells. These cells are known to divide every 2 to 5 days, replacing cells that slough off the tips of the villi in a continual process [20]. LT treatment reduced cellular proliferation in the villous crypts up to 48 h following exposure (**Figure 5**, upper middle panel), and this was associated with a breakdown of the normal villous structure. Administration of a protease-deficient mLT had no effect on baseline proliferation in the intestinal crypts (**Figure 5**, upper right panel). Staining with anti-cleaved (activated) caspase-3 revealed marked apoptosis in the villous tips of LT-treated animals (**Figure 5**, lower middle panel), compared to the scant staining observed in samples from PBS-treated (**Figure 5**, lower left panel) or mLT-treated animals (**Figure 5**, lower right panel). Taken together, these data establish that the specific proteolytic activity of LF on the MKKs is required for the anti-proliferative and pro-apoptotic effects of LT on the intestinal epithelium. As the blockade in intestinal cell proliferation and the evidence of enhanced caspase-3-mediated apoptosis correlate temporally with the observed intestinal damage, the findings support a role for these mechanisms in mediating the pathological effects of LT on the intestine.

## Discussion

Recent research efforts have focused on inhalation as a route of anthrax infection, which is the preferred route for intentional exposure of humans when using anthrax as a weapon [21], [22], rather than the gastrointestinal route of infection that characterizes the natural epidemiology of *Bacillus anthracis* infection [23]. Presumably, *Bacillus anthracis* has evolved strategies to overcome host defenses at the most common initial site of host penetration, the intestinal tract. Our studies have extensively characterized the timing and pattern of LT-induced intestinal pathology, which is marked by villous blunting, mucosal erosions, and ulceration. These structural defects, when combined with the well-described immunosuppressive effects of LT [24], [25] lead to systemic infections with enteric bacteria that would otherwise exist in a commensal relationship with their mammalian hosts. LT-induced intestinal damage and infectious sequelae require the proteolytic activity of its LF component, highlighting the essential role of MKK-dependent signaling in maintaining the integrity of the intestinal barrier. It should be noted that mice treated with antibiotics in combination with LT had a similar mortality curve to those receiving LT alone (data not shown), indicating that LT-induced infection is not required for the toxin to cause mortality as a stand-alone agent; other toxin-mediated effects are sufficient to mediate lethality. Nevertheless, these results are relevant in the setting of infection, exposing a potential toxin-mediated mechanism that could serve to promote bacterial entry via the intestinal route.

In addition, progress has been made toward identifying the mechanisms that underlie the enterotoxic effects of LT. In this regard, the LT-induced proliferative block observed in intestinal epithelial progenitor cells would be predicted to disrupt the normal cycle of displacement/replacement of terminally differentiated enterocytes, thereby accounting for the observed accumulation of apoptotic cells at the villous tips, accompanied by signs of villous blunting and ulceration. Subsequent expansion of the intestinal ulcers depends partially on bacterial activity, as antibiotics attenuate ulcer progression. That LT-induced immunosuppression contributes to bacterial invasion and dissemination appears a likely hypothesis and is consistent with findings of intestinal pathology in immunosuppressed MyD88 ^−/−^ mice, but not MyD88 ^+/−^ mice, treated with a lower dose of LT [11]. In contrast to radiation- and chemotherapy-induced immunosuppression that is maximal when neutrophil counts drop days following exposure [13], [14], [15], the immunosuppressive effect of anthrax LT on MKK signaling occurs in hours [26], [27], [28], correlating with the relatively rapid development of the intestinal syndrome in LT-exposed mice. Nevertheless, other potential pathogenic mechanisms should be considered as well. LT alters the cellular localization and trafficking of key junction associated proteins, such as the cadherins and ZO1, resulting in dysregulation of the junctional complexes that establish functional cellular barriers [29], [30]. Studies addressing a potential role for these pathways in mediating the effects of LT on the intestine are warranted. Moreover, the development of a rodent model for gastrointestinal anthrax involving toxigenic *Bacillus anthracis* would advance progress in this field as well; currently, this experimental tool is not available to the scientific community [31].

Our experimental findings further define the critical host targets of LT, as well as clarify inconsistencies in the literature. First, we observed that the enterotoxic effect of LT is dose-dependent. Administration of a decreased dose of LT results in mortality, but without concomitant intestinal pathology, indicating that differences in the dose and/or potency of LT likely accounted for previous reports of minimal LT-induced intestinal pathology in wild-type hosts. Second, although LT drives the breakdown of the intestinal barrier and resultant enteric bacterial infections, the effects of LT on other host targets account for its lethality when administered parenterally as a stand-alone agent. More importantly, these results firmly establish MKKs as critical regulators of the interface between the host and potential pathogens in the intestinal tract. Proteolysis and inactivation of MKKs by anthrax LT induces intestinal ulceration and the subsequent development of disseminated infection with enteric organisms. The rapidity (48–72 h) and severity of this effect matches or exceeds that associated with radiation or chemotherapy injury [13], [14], [15], [16]. These findings not only have direct ramifications for the pathogenic mechanisms of *Bacillus anthracis*, but they suggest intriguing implications for enteric pathogens known to target MKK/MAPK pathways via specific virulence factors [6], [7], [8], [9].

## Materials and Methods

### Animal strains

Experiments involved mice that were treated in accordance with an animal protocol (#WO2006-58), which was approved by the CBER Institutional Animal Care and Use Committee. Experiments involved female BALB/c and C57BL/6J mice that were 8–12 weeks of age, which were purchased from The Jackson Laboratory. Mice were allowed at least one week to acclimatize to the animal facilities prior to experimentation.

### Western blotting

Murine protein extracts were prepared using T-PER Tissue Protein Extraction Reagent (Thermo Scientific, Rockford, IL), following the manufacturer's suggested protocol. Protein samples were electrophoretically separated on 4–12% NuPage Bis-Tris gradient gels (Invitrogen, Carlsbad, CA) and transferred to 0.45-µm nitrocellulose membranes. The membranes were incubated with the following primary antibodies at the indicated concentrations: anti-mitogen activated protein kinase (MKK)-3, 200 ng/ml (Santa Cruz Biotechnology, Santa Cruz, CA; sc-959); anti-MKK-4, 100 ng/ml (Santa Cruz Biotechnology, Santa Cruz, CA; sc-964) or anti-β-actin 200 ng/ml (Sigma, St. Louis, MO; A2228). After incubation with the appropriate species-specific HRP-conjugated secondary antibodies, the membranes were then incubated with SuperSignal West Pico chemiluminescent substrate (Thermo Scientific, Rockford, IL) and exposed to autoradiograph film.

### *In vivo* intoxication experiments

Lyophilized recombinant PA, LF, and mutant LF (E687C) were purchased from List Biological Laboratories, Inc. (Campbell, CA) and reconstituted in sterile water (stock concentration: 1 mg/mL in 5 mM HEPES, 50 mM NaCl, pH 7.5). Anthrax LT was administered with a fixed ratio of PA/LF of 2.5∶1 by weight (molar ratio: 2.7∶1). Unless otherwise indicated, mice were injected intravenously with 200 µg PA/80 µg LF (C57BL/6J) or 100 µg PA/40 µg LF (BALB/c), administered in a total volume of 0.3 mL of PBS. As a negative control, selected mice received the same volume of PBS alone. Mice were sacrificed when moribund or as otherwise indicated. Blood samples were collected aseptically either by cardiac puncture (**Figure 4B**) or from the tail vein (**Figure S1**). Blood glucose levels were determined using an automatic glucose analyzer (Ascensia Elite XL, Bayer). Central blood glucose concentrations in cardiac puncture samples were consistently higher than concentrations detected in tail vein samples.

Some experiments involved the concomitant administration of broad-spectrum antibiotics with anthrax LT. Both antibiotics were administered subcutaneously beginning the day of LT treatment (amoxicillin: 100 mg/kg every 8 hours, and gentamicin 16 mg/kg one time per day, Sigma, St. Louis, MO). Antibiotic therapy was maintained until the animal succumbed to toxemia or until the termination of the experiment.

Gross pathological assessments were performed on dissected murine gastrointestinal tracts, which were photographed against a white background using a Nikon D40 camera.

### Histological assessments

To evaluate the intestinal damage caused by anthrax LT or protease-deficient mutant LT, intestinal samples were collected from each mouse and fixed in 10% neutral buffered formalin (Sigma, St. Louis, MO). Paraffin sectioning, hematoxylin and eosin staining (H&E), and Brown and Brenn (B&B) staining were performed by Histoserv, Inc (Gaithersburg, MD). In addition, Histoserv performed immunohistochemistry staining using the following antibodies: anti-Ki67 (Abcam, Cambridge, MA, Ab16667, dilution: 1∶100) and anti-cleaved caspase-3 (Cell Signaling Technology, Danvers, MA; #9664, dilution: 1∶400). H&E-, B&B- and immunohistochemistry-stained slides were scanned using an Aperio ScanScope (ScanScope, Aperio, CA). The images were acquired at 20× magnification, and relevant areas were converted into a TIF format at various magnifications for the generation of figures. Stained tissue sections were analyzed by a board-certified veterinary pathologist in a blinded fashion.

### Bacterial isolation and identification

Bacterial isolates were cultured from aseptically-obtained blood (cardiac puncture) or peritoneal wash samples using blood agar plates (Remel, Lenexa, KS; RO1202) and/or MacConkey plates (Remel, Lenexa, KS; RO1552). Peritoneal cavity washes were performed using 3 mL of PBS; 100 µL of the post-wash samples was cultured on blood agar plates. Bacterial identifications were performed by the NIH Department of Laboratory Medicine, using the Siemens Micro Scan Auto SCAN4 system (Deerfield, IL), supplemented with conventional biochemical tests [32], [33]. To quantify bacteria in blood or peritoneal wash specimens, samples were serially diluted and cultured on blood agar plates prior to overnight culture and enumeration.

## Supporting Information

Figure S1 **LT causes a drop in serum glucose concentrations** ***in vivo.*** C57BL/6J mice were injected intravenously with LT (n = 15) or PBS (n = 15). Tail vein blood samples were assessed for glucose concentration at varying time points following administration as shown. (* p<0.001, ** p<0.0001, Student's t-test).
(TIF)

Table S1 **Bacterial Culture Results at Autopsy (BALB/c).**
(DOC)

Table S2 **Bacterial Culture Results at Autopsy.**
(DOC)

Table S3 **Bacterial Culture Results at Autopsy (C57BL/6J Mice).**
(DOC)

Table S4 **Effect of Antibiotics on LT-induced Bacteremia in C57BL/6J Mice.**
(DOC)

## References

[1] Fasanella A, Galante D, Garofolo G, Jones MH (2010). Anthrax undervalued zoonosis.. Vet Microbiol.

[2] Kolsto AB, Tourasse NJ, Okstad OA (2009). What sets Bacillus anthracis apart from other Bacillus species?. Annu Rev Microbiol.

[3] Young JA, Collier RJ (2007). Anthrax toxin: receptor binding, internalization, pore formation, and translocation.. Annu Rev Biochem.

[4] Moayeri M, Leppla SH (2009). Cellular and systemic effects of anthrax lethal toxin and edema toxin.. Mol Aspects Med.

[5] Xie T, Auth RD, Frucht DM (2011). The Effects of Anthrax Lethal Toxin on Host Barrier Function.. Toxins.

[6] Arbibe L, Kim DW, Batsche E, Pedron T, Mateescu B (2007). An injected bacterial effector targets chromatin access for transcription factor NF-kappaB to alter transcription of host genes involved in immune responses.. Nat Immunol.

[7] Mittal R, Peak-Chew SY, McMahon HT (2006). Acetylation of MEK2 and I kappa B kinase (IKK) activation loop residues by YopJ inhibits signaling.. Proc Natl Acad Sci U S A.

[8] Lin SL, Le TX, Cowen DS (2003). SptP, a Salmonella typhimurium type III-secreted protein, inhibits the mitogen-activated protein kinase pathway by inhibiting Raf activation.. Cell Microbiol.

[9] Shan L, He P, Sheen J (2007). Intercepting host MAPK signaling cascades by bacterial type III effectors.. Cell Host Microbe.

[10] Moayeri M, Haines D, Young HA, Leppla SH (2003). Bacillus anthracis lethal toxin induces TNF-alpha-independent hypoxia-mediated toxicity in mice.. J Clin Invest.

[11] Okugawa S, Moayeri M, Eckhaus MA, Crown D, Miller-Randolph S (2011). MyD88-dependent signaling protects against anthrax lethal toxin-induced impairment of intestinal barrier function.. Infect Immun.

[12] Fang H, Sun C, Xu L, Owen RJ, Auth RD (2010). Neutrophil elastase mediates pathogenic effects of anthrax lethal toxin in the murine intestinal tract.. J Immunol.

[13] Dainiak N, Waselenko JK, Armitage JO, MacVittie TJ, Farese AM (2003). The hematologist and radiation casualties.. Hematology Am Soc Hematol Educ Program.

[14] Schuller BW, Binns PJ, Riley KJ, Ma L, Hawthorne MF (2006). Selective irradiation of the vascular endothelium has no effect on the survival of murine intestinal crypt stem cells.. Proc Natl Acad Sci U S A.

[15] Khan SA, Wingard JR (2001). Infection and mucosal injury in cancer treatment.. J Natl Cancer Inst Monogr.

[16] Brook I, Elliott TB, Ledney GD, Knudson GB (2002). Management of postirradiation sepsis.. Mil Med.

[17] Boyden ED, Dietrich WF (2006). Nalp1b controls mouse macrophage susceptibility to anthrax lethal toxin.. Nat Genet.

[18] Rink RD, Short BL, Van Van N, Fry DE (1977). Role of colonic bacteria in the pathophysiology of fecal peritonitis.. Circ Shock.

[19] Miller SI, Wallace RJ, Musher DM, Septimus EJ, Kohl S (1980). Hypoglycemia as a manifestation of sepsis.. Am J Med.

[20] Tian H, Biehs B, Warming S, Leong KG, Rangell L (2011). A reserve stem cell population in small intestine renders Lgr5-positive cells dispensable.. Nature.

[21] Jernigan DB, Raghunathan PL, Bell BP, Brechner R, Bresnitz EA (2002). Investigation of bioterrorism-related anthrax, United States, 2001: epidemiologic findings.. Emerg Infect Dis.

[22] Franz DR (2009). Preparedness for an anthrax attack.. Mol Aspects Med.

[23] Turnbull PC (2002). Introduction: anthrax history, disease and ecology.. Curr Top Microbiol Immunol.

[24] Xu L, Frucht DM (2007). Bacillus anthracis: a multi-faceted role for anthrax lethal toxin in thwarting host immune defenses.. Int J Biochem Cell Biol.

[25] Tournier JN, Rossi Paccani S, Quesnel-Hellmann A, Baldari CT (2009). Anthrax toxins: a weapon to systematically dismantle the host immune defenses.. Mol Aspects Med.

[26] Fang H, Cordoba-Rodriguez R, Lankford CS, Frucht DM (2005). Anthrax lethal toxin blocks MAPK kinase-dependent IL-2 production in CD4+ T cells.. J Immunol.

[27] Fang H, Xu L, Chen TY, Cyr JM, Frucht DM (2006). Anthrax lethal toxin has direct and potent inhibitory effects on B cell proliferation and immunoglobulin production.. J Immunol.

[28] Xu L, Fang H, Frucht DM (2008). Anthrax lethal toxin increases superoxide production in murine neutrophils via differential effects on MAPK signaling pathways.. J Immunol.

[29] Warfel JM, Steele AD, D'Agnillo F (2005). Anthrax lethal toxin induces endothelial barrier dysfunction.. Am J Pathol.

[30] Guichard A, McGillivray SM, Cruz-Moreno B, van Sorge NM, Nizet V (2010). Anthrax toxins cooperatively inhibit endocytic recycling by the Rab11/Sec15 exocyst.. Nature.

[31] Glomski IJ, Piris-Gimenez A, Huerre M, Mock M, Goossens PL (2007). Primary involvement of pharynx and peyer's patch in inhalational and intestinal anthrax.. PLoS Pathog.

[32] Murray P, Baron E, Jorgensen J, Landry M, Pfaller M (2007). Manual of Clinical Microbiology.

[33] Kiratisin P, Li L, Murray PR, Fischer SH (2003). Identification of bacteria recovered from clinical specimens by 16S rRNA gene sequencing.. Eur J Clin Microbiol Infect Dis.

