# Supplementary figures and images for: Anthrax Lethal Toxin Disrupts Intestinal Barrier Function and Causes Systemic Infections with Enteric Bacteria
Source: PLoS One. 2012 Mar 16;7(3):e33583. doi: 10.1371/journal.pone.0033583 (PMC3306423; doi:10.1371/journal.pone.0033583)

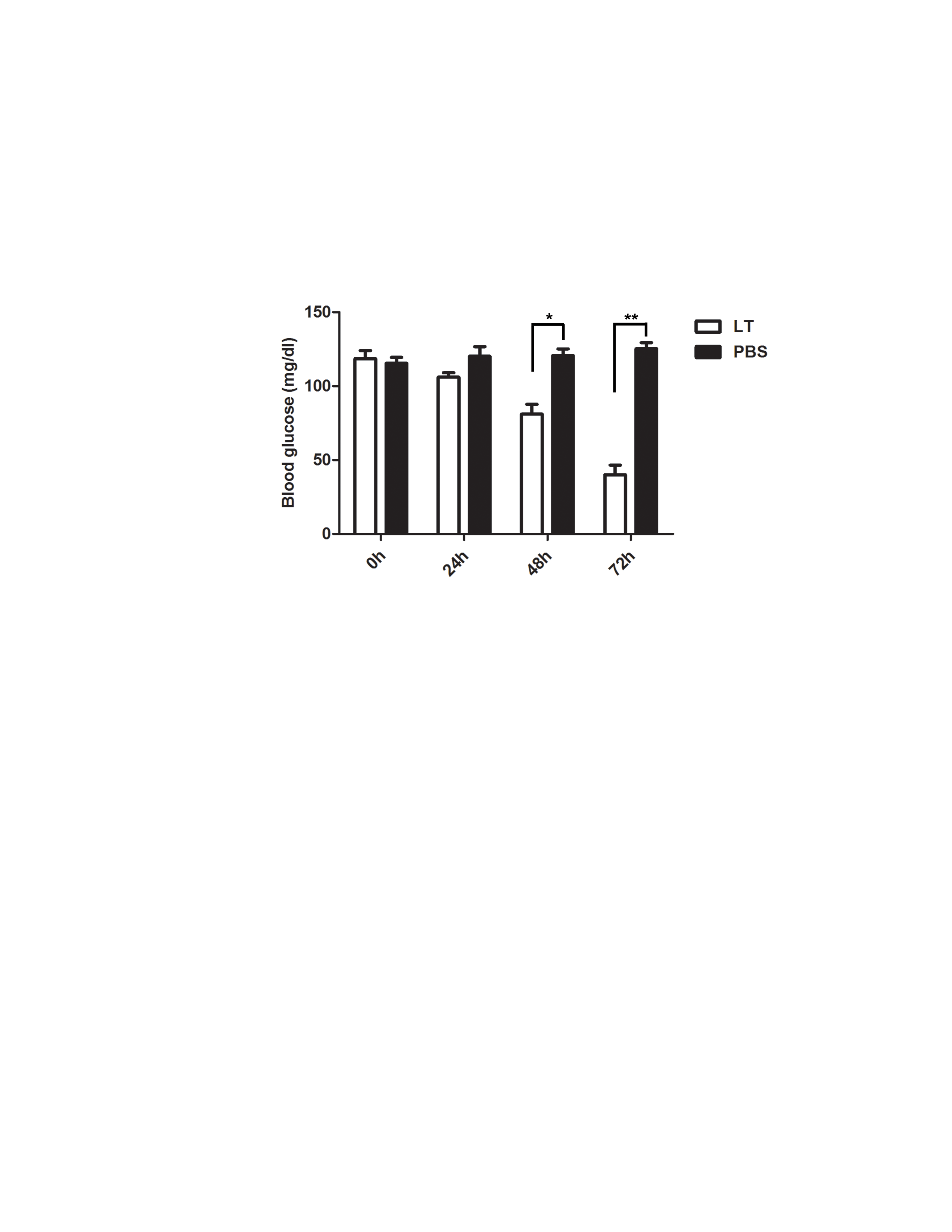

Supplement: Figure S1 — LT causes a drop in serum glucose concentrations in vivo. C57BL/6J mice were injected intravenously with LT (n = 15) or PBS (n = 15). Tail vein blood samples were assessed for glucose concentration at varying time points following administration as shown. (* p<0.001, ** p<0.0001, Student's t-test). (TIF) [file pone.0033583.s001.tif]
