# Supplementary material for: Anthrax Lethal Toxin Disrupts Intestinal Barrier Function and Causes Systemic Infections with Enteric Bacteria
Source: PLoS One. 2012 Mar 16;7(3):e33583. doi: 10.1371/journal.pone.0033583 (PMC3306423; doi:10.1371/journal.pone.0033583)
Supplement: Table S1 — Bacterial Culture Results at Autopsy (BALB/c). (DOC) [file pone.0033583.s002.doc]

**Table S1. Bacterial Culture Results at Autopsy (BALB/c)**

|  | **Bacterial Culture Results** | |
| --- | --- | --- |
| **n** | **Abdominal cavity** | **Blood** |
| 9 | **** | **** |
| 3* | **+** | **** |
| 0 | **** | **+** |
| 2* | **+** | **+** |

* The five BALB/c mice that developed systemic infection survived 52-66 hours post LT exposure.
