# Supplementary material for: Anthrax Lethal Toxin Disrupts Intestinal Barrier Function and Causes Systemic Infections with Enteric Bacteria
Source: PLoS One. 2012 Mar 16;7(3):e33583. doi: 10.1371/journal.pone.0033583 (PMC3306423; doi:10.1371/journal.pone.0033583)
Supplement: Table S2 — Bacterial Culture Results at Autopsy. (DOC) [file pone.0033583.s003.doc]

**Table S2. Bacterial Culture Results at Autopsy**

|  | **C57BL/6J** | | | **BALB/c** | | |
| --- | --- | --- | --- | --- | --- | --- |
|  | **PBS (n=5)** | **mLF (n=5)** | **LF (n=5)** | **PBS (n=5)** | **mLF (n=5)** | **LF (n=5)** |
| Culture positive in abdominal cavity | 0 | 0 | 4 | 0 | 0 | 2 |
| Bacteremia | 0 | 0 | 5 | 0 | 0 | 1 |
