# Supplementary material for: Anthrax Lethal Toxin Disrupts Intestinal Barrier Function and Causes Systemic Infections with Enteric Bacteria
Source: PLoS One. 2012 Mar 16;7(3):e33583. doi: 10.1371/journal.pone.0033583 (PMC3306423; doi:10.1371/journal.pone.0033583)
Supplement: Table S3 — Bacterial Culture Results at Autopsy (C57BL/6J Mice). (DOC) [file pone.0033583.s004.doc]

**Table S3. Bacterial Culture Results at Autopsy (C57BL/6J Mice)**

|  | **Lethal toxin** | | |
| --- | --- | --- | --- |
|  | **PBS (n=5)** | **low dose (n=10)** | **high dose (n=10)** |
| Culture positive in abdominal cavity | 0 | 3 | 10 |
| Bacteremia | 0 | 4 | 9 |
