# Supplementary material for: Anthrax Lethal Toxin Disrupts Intestinal Barrier Function and Causes Systemic Infections with Enteric Bacteria
Source: PLoS One. 2012 Mar 16;7(3):e33583. doi: 10.1371/journal.pone.0033583 (PMC3306423; doi:10.1371/journal.pone.0033583)
Supplement: Table S4 — Effect of Antibiotics on LT-induced Bacteremia in C57BL/6J Mice. (DOC) [file pone.0033583.s005.doc]

**Table S4. Effect of Antibiotics on LT-induced Bacteremia in C57BL/6J Mice**

|  | PBS (n=15) | Lethal toxin + PBS (n=15) | Lethal toxin + Antibiotics (n=15) |
| --- | --- | --- | --- |
| Culture positive in abdominal cavity | 0 | 14 | 1 |
| Bacteremia | 0 | 15 | 0 |
